# Identification, Sequencing, and Molecular Analysis of RNA2 of Artichoke Italian Latent Virus Isolates from Known Hosts and a New Host Plant Species

**DOI:** 10.3390/v15112170

**Published:** 2023-10-28

**Authors:** Toufic Elbeaino, Amani Ben Slimen, Imen Belgacem, Monia Mnari-Hattab, Roberta Spanò, Michele Digiaro, Ahmed Abdelkhalek

**Affiliations:** 1 Istituto Agronomico Mediterraneo of Bari, Via Ceglie 9, 70010 Valenzano, Italy; b.slimen@iamb.it (A.B.S.); imen.belgacem@univ-brest.fr (I.B.); digiaro@iamb.it (M.D.); 2 Laboratoire de Biotechnologie Appliquée à l’Agriculture, INRA Tunisie, Université de Carthage, Rue Hedi Karray, Tunis 1004, Tunisia; 3 Dipartimento di Scienze del Suolo, della Pianta e degli Alimenti, Università degli Studi di Bari, Via G. Amendola, 165/A, 70126 Bari, Italy; roberta.spano@uniba.it; 4 Plant Protection and Biomolecular Diagnosis Department, ALCRI, City of Scientific Research and Technological Applications, New Borg El Arab City 21934, Egypt; abdelkhalek2@yahoo.com

**Keywords:** nepovirus, RNA2 sequences, phylogenetic and recombination analyses

## Abstract

Despite its first description in 1977 and numerous reports of its presence in various plant species in many countries, the molecular information available in GenBank for artichoke Italian latent virus (AILV) is still limited to a single complete genome sequence (RNA1 and 2) of a grapevine isolate (AILV-V) and a partial portion of the RNA2 sequence from an isolate of unknown origin and host. Here, we report the results of molecular analyses conducted on the RNA2 of some AILV isolates, sequenced for the first time in this study, together with the first-time identification of AILV in a new host plant species, namely chard (*Beta vulgaris* subsp. vulgaris), associated with vein clearing and mottling symptoms on leaves. The different AILV isolates sequenced were from artichoke (AILV-C), gladiolus (AILV-G), Sonchus (AILV-S), and chard (AILV-B). At the molecular level, the sequencing results of the RNA2 segments showed that AILV-C, AILV-G, AILV-S, and AILV-B had a length of 4629 nt (excluding the 3′ terminal polyA tail), which is one nt shorter than that of the AILV-V reported in GenBank. A comparison of the RNA2 coding region sequences of all the isolates showed that AILV-V was the most divergent isolate, with the lowest sequence identities of 83.2% at the nucleotide level and 84.7% at the amino acid level. Putative intra-species sequence recombination sites were predicted among the AILV isolates, mainly involving the genomes of AILV-V, AILV-C, and AILV-B. This study adds insights into the variability of AILV and the occurrence of recombination that may condition plant infection.

## 1. Introduction

The genus *Nepovirus*, family *Secoviridae*, was one of the first group of plant viruses recognized by the International Committee on Taxonomy of Viruses (ICTV) [1,2]. Nepoviruses have a bipartite positive single-stranded RNA genome (RNA1 and RNA2) and are divided into three subgroups (A, B, and C) based on their sequences, genome organization, and cleavage sites [3]. Currently, this genus includes 48 recognized viruses [4], of which artichoke Italian latent virus (AILV) belongs to subgroup B [4]. Moreover, based on RNA2 size, nepoviruses of subgroups A, B, and C show different virus particle sediment properties: subgroup A has viruses with an RNA2 of 3.7–4.0 Kb, present in both middle (M) and bottom (B) components; subgroup B has viruses with an RNA2 of 4.4–4.7 Kb, present only in the M component; and subgroup C has an RNA2 of 6.4–7.3 Kb, present in M component particles that are sometimes barely separable from those of the B component [5]. 

After the first discovery of AILV in Italy in asymptomatic artichoke plants (*Cynara scolymus* L.) [6], this virus was found in Bulgaria in several plant species: chicory (*Cichorium intybus*) with leaf chlorotic mottle and yellow spots [7]; pelargonium (*Pelargonium zonale*) with severe leaf malformations and stunting symptoms [8]; sow thistle (*Sonchus oleraceus*) with yellow rings and line pattern on leaves [9]; gladiolus (*Gladiolus palustris*); and grapevine (*Vitis vinifera*) with fanleaf-like symptoms and reduced growth [10,11]. AILV was also found in several weeds (i.e., *Crepis neglecta*, *Helminthia chioides*, *Hypochaeris aetnensis*, *Lactuca virosa*, *Urospermum dalechampii*, and *Lamium amplexicaule*) [12].

A comparative analysis conducted on three Bulgarian AILV isolates from sow thistle (AILV-S), gladiolus (AILV-G), and grapevine (AILV-V) and one Italian isolate from artichoke (AILV-C) showed that all share common biological, serological, and physical–chemical properties [10]. At the molecular level, only recently has the genome of AILV-V been completely sequenced [13], whereas no sequence information is available from other isolates, including those reported on weeds. 

Here, we report the molecular information (sequence identities, genetic variability, recombination, and phylogenetic relationship), limited to RNA2, from the genomes of still non-sequenced AILV isolates (AILV-C, G, and S) and of a new isolate (AILV-B) found for the first time in this study in chard plants (*Beta vulgaris* subsp. *vulgaris*). The information gained in this study was obtained through the application of serial biological (mechanical inoculation) and molecular (RT-PCR, “primer walking” strategy, and sequencing) experiments conducted on the RNA-2 segment of each isolate.

Furthermore, since recombination is recurrent among nepoviruses [14,15,16,17,18,19,20], this aspect was investigated in the genomic RNA2 sequences of all the AILV isolates, the results of which are reported and discussed here.

## 2. Materials and Methods

### 2.1. Source of Plant Material

The AILV isolates used in this study originated from the lyophilized leaves of different *Nicotiana occidentalis* plants that were initially inoculated in one single transmission step from their original hosts and conserved at the IPSP-CNR Institute of Bari [10]. 

The AILV-B isolate, on the other hand, was identified by chance in some chard plants, adjacent to an artichoke field in Bizerte (northern Tunisia), whose nucleic acids were initially thought to be used as negative controls in dot blot hybridization assays during a survey for AILV in artichoke plants [21,22].

All the AILV isolates, including the one found in chard plants (AILV-B) (see details below), were sap-inoculated onto *N. occidentalis* plants by grinding 1 g of lyophilized plant material in a mortar with 5 mL of 0.1 mM of phosphate buffer (pH 7.3). In total, 20 *N. occidentalis* plants were used for each AILV isolate, and at the onset of symptoms (8 days post inoculation, DPI), 10–15 plants (ca. 50 g) were used for purification following fractionation in 10–40% linear sucrose density gradients [10].

### 2.2. Extraction of Total Nucleic Acids, cDNA Synthesis, and PCR 

Nucleic acids were extracted from 1µg of purified virus preparations after incubation with 1% SDS and the addition of 1 vol Tris-EDTA-saturated phenol and chloroform [23]. The two RNA segments of AILV were electrophoresed in 1.5% agarose gel in 1x TBE buffer and stained with GelRed nucleic acid stain (Biotium, Rome, Italy). cDNA was synthesized using 0.5 µg of RNAs and an equal quantity of random DNA hexanucleotide mixture and of an oligodT primer for the RT-PCR amplifications of internal genomic portions and the 5′ end of the genomic RNA2 of AILV, respectively. The *Moloney murine leukemia virus* enzyme (200 units) was used in reverse transcription in a 20 µL reaction for 1 h at 42 °C, following the manufacturer’s instructions (LifeTechnologies, Milan, Italy). 

A set of sense and antisense primers (Supplementary Table S1), designed on an RNA2 sequence of an AILV-V isolate from GenBank (acc.no. LT608396), were initially used in the RT-PCR and 5′\3′ RACE-PCR assays to amplify the corresponding viral genomic regions of all the AILV isolates. Subsequently, where amplification failed, new primer sets were designed on the newly obtained sequences (as a “primer walking” strategy) to close the sequence gaps between the missing fragments. All the RT-PCR runs consisted of 40 cycles, with an initial denaturing temperature of 94 °C for 1 min, 58 °C for 30 sec, and an elongation time of 1 min at 72 °C. The RT-PCR products were electrophoresed in 1.2% agarose gel in 1x TAE buffer and stained with GelRed nucleic acid stain.

### 2.3. Cloning and Sequencing

All the RT-PCR amplicons were ligated into StrataClone^TM^ PCR cloning vector pSC-A (Stratagene, CA, USA), subcloned into *Escherichia coli* DH5α and custom sequenced (Eurofins Genomics, Germany). The nucleotide and protein sequences were analyzed (10 July 2023) using Geneious Prime vers. 2023.2 (https://www.geneious.com, access date: 10 July 2023, Auckland, New Zealand). The Blast programs (i.e., BlastX and BlastP) of the NCBI (National Center for Biotechnology Information) were used to search for homologies in the protein information resources’ databases (PIR, release 47.0) [24]. A tentative phylogenetic tree was constructed using the neighbor-joining (NJ) method in the Geneious Prime vers. 2023.2 analysis package. A bootstrap value for each node of NJ trees was calculated using 1000 replicates.

### 2.4. Sequence and Recombination Analyses

The RNA2 sequences obtained were analyzed for genetic variability and possible recombination using the “Recombination Detection Program” (RDP4) version 4.22, with default parameters (the highest acceptable probability value = 0.05). The RDP4 package uses several programs to detect the occurrence of robust recombination events, namely RDP, GENECONV, BOOTSCAN, MAXCHI, CHIMAERA, 3SEQ, and SISCAN. Recombination sites identified by four or more programs and two or more types of methods were considered to be “significant recombination events”.

## 3. Results

### 3.1. Complete Sequence of Genomic RNA2 of AILV Isolates

All the sequences derived from the RACE- and RT-PCR-generated amplicons were analyzed using the sense and antisense AILV-specific primers, and the complete RNA2 sequences were constructed. The RNA2 sequences of AILV-C (LT608397), AILV-G (LT608398), and AILV-S (MT294111) were found to be 4629 nt long, 1 nucleotide shorter than AILV-V (LT608396), which contained 1 nt more in the 3′UTR region. 

The ORF2 of all the AILV isolates extended from nt 289 to 4332 and coded for a polyprotein (p2) with a predicted molecular mass (Mr) of ca. 149.5 KDa, comprising, in the 5′→ 3′ direction, the homing protein (2A^HP^), the movement protein (2B^MP^), and the coat protein (2C^CP^) domains. All the AILV isolates except AILV-C (R/A) showed a dipeptide (K/A) at aa position 835-836, which is also reported to be a cleavage site for TBRV CP [17], cleaving the MP and the CP domains. However, for subgroup B nepoviruses, the cleavage site between HP and MP is still uncertain, although the hypothetical site (M/A) has recently been predicted to cleave these domains [25].

### 3.2. Identification of AILV in Chard Plants

Surprisingly, two out of seven harvested chard plants yielded positive reactions. To confirm this unexpected result, all seven chard plants were subjected to a new RT-PCR assay using a couple of AILV-specific primer pairs, i.e., F1s\F1a and F2s\F2a, which amplify two different portions of RNA2, of 330 bp and 420 bp, respectively (see Supplementary Table S1). Both RT-PCR assays confirmed the AILV detection in the two dot blot hybridization-positive samples and in two other samples. The RT-PCR amplicons of the AILV isolates from the four infected chard plants (AILV-B) were sequenced. For one of these, sequencing was extended to the entire RNA2 (MT294112), which was then analyzed together with those obtained from other plant species.

### 3.3. Mechanical Transmission of AILV Chard Plants onto Herbaceous Host

**Figure 1:**
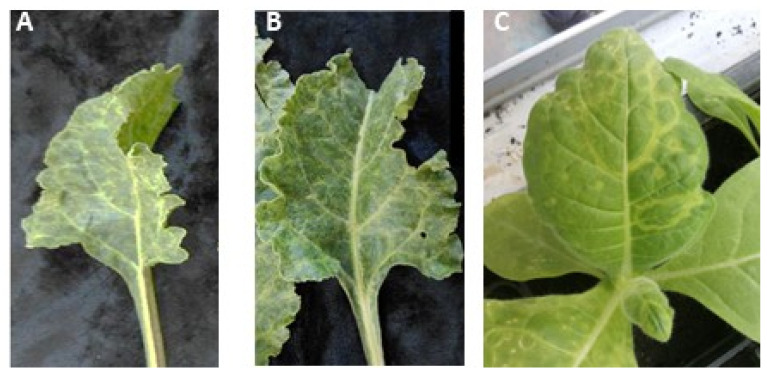
Leaves of two chard plants (**A**, **B**) infected with AILV and showing vein clearing and mottling symptoms. Leaves of an *N. occidentalis* plant (**C**), mechanically inoculated with AILV-infected chard sap, showing chlorotic ringspot and vein mottling symptoms typical of nepovirus infections.

Sap extracted from the two AILV-infected chard plants, showing mottling and vein clearing symptoms and that were positive in both RT-PCR and dot blot hybridization assays, was used to transmit AILV to *N. occidentalis* plants. Eight days after inoculation, the mechanically inoculated plants showed ringspots and vein mottling symptoms (Figure 1). The absence in *N. occidentalis* of other common chard-infecting viruses, i.e., cucumber mosaic virus (CMV) [26], beet curly top virus (BCTV) [27], beet mosaic virus (BtMV) [28], beet yellows virus (BYV) [29], turnip mosaic virus (TuMV) [30], and cauliflower mosaic virus (CaMV) [31], was ascertained via RT-PCR and real-time PCR assays; thus, the symptoms that developed on *N. occidentalis* are probably attributable to AILV infection. 

### 3.4. Genetic Variability of Genomic RNA2 of AILV Isolates 

Between the 5′ and 3′UTRs of RNA2 of all the AILV isolates under study there were different levels of nucleotide variation (Table 1), which were particularly high for the AILV-B isolate on both segments. 

The comparative sequence analysis between the RNA2 coding region of all the isolates showed that AILV-V was the most divergent isolate, with sequence identities of 83.2% at the nucleotide level and 84.7% at the amino acid level (Table 2).

At the coat protein (CP) level, the sequence variability among the AILV isolates was in line with that observed in other nepoviruses and was, in any case, below the demarcation threshold of nepoviruses of 25% at the amino acid level [32]. The greatest variability was found in AILV-V and AILV-C with a 12.7% difference at the nt level and 5.1% at the aa level (Table 3). 

However, the variability within the CP gene increased when the sequence of an AILV isolate of unknown origin and host species, reported in the GenBank (X28754), was included in the comparative analysis. 

**Figure 2:**
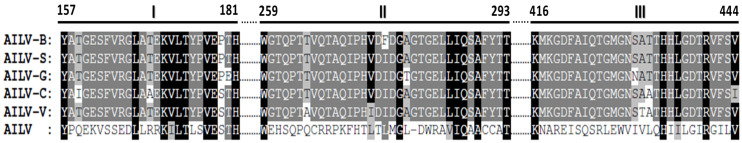
Amino acids alignment of the five 2C^CP^ gene of the AILV isolates sequenced in this study (AILV-B, -S, -G, -C, and -V) and that reported in the GenBank (AILV: X28754), showing the positions of major variabilities (I, II, and III). Regions with high homology are highlighted in gray. Conserved amino acid residues in the six isolates are highlighted in black. Numbers correspond to the position of the amino acids in the CP gene.

In particular, the sequence alignment of the 2C^CP^ gene of the five AILV isolates and AILV-X28754 showed three non-conserved aa stretches that were completely different from all the AILV sequences under study (Figure 2), while variability was 15.2% at the nt level and 20% at the aa level.

### 3.5. Phylogenetic Analysis of AILV Isolates 

**Figure 3:**
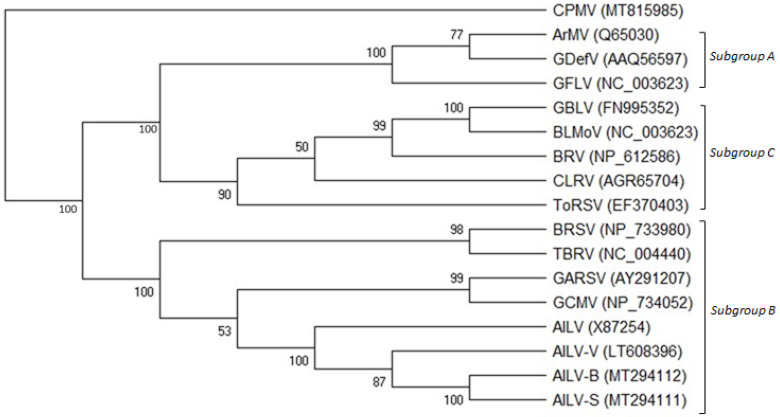
Phylogenetic tree generated from alignment of the complete RNA-2 polyprotein amino acid sequences of members belonging to subgroups A, B, and C of the genus *Nepovirus*. GenBank accession numbers of the sequences used are shown in brackets. The viruses used in the phylogenetic analysis are the following: grapevine chrome mosaic virus (GCMV), grapevine Anatolian ringspot virus (GARSV), artichoke Italian latent virus isolates (AILV-S, -B, -G, -C, and -V), tomato black ring virus (TBRV), beet ringspot virus (BRSV), grapevine deformation virus (GDefV), arabis mosaic virus (ArMV), grapevine fanleaf virus (GFLV), blueberry leaf mottle virus (BLMoV), grapevine Bulgarian latent virus (GBLV), blackcurrant reversion virus (BRV), tomato ringspot virus (ToRSV), and cherry leafroll virus (CLRV). Cowpea mosaic virus (CPMV) was used as an outgroup species. Bootstrap values above 50% are shown at branch points (1000 replicates).

The phylogenetic tree constructed on the amino acid sequence alignment of the 2C^CP^ genes of all the AILV isolates and those of other nepoviruses of subgroups A, B, and C allocated the AILV isolates into three clusters within a clade composed of viruses belonging to subgroup B; AILV-V and AILV-X87254 were allocated into two clusters separated from all the other isolates (Figure 3).

### 3.6. Recombination Analysis

The recombination analysis conducted on the RNA2 sequences of the AILV isolates generated high P-values, at least in five implemented methods, which were taken as indicators of significant intraspecific recombination sites occurring in different regions of the genomic RNA of the AILV isolates (Table 4).

## 4. Discussion and Conclusions

This study aimed to provide additional molecular information on the subgroup B nepovirus AILV by analyzing the genome sequences of AILV isolates infecting different host species, to be added to the only complete genome sequence (RNA1 and 2) of a grapevine AILV isolate present in GenBank. The complete sequencing of five RNA2 coding region segments of AILV from different host species showed the presence of a high genomic variability (ca. 17.8% at nt level and 14.1% aa level). Another highlight of this study was the first-time finding of AILV in chard (*Beta vulgaris*) plants, associated with vein mottling and yellowing. Unfortunately, it was not possible to corroborate this result further by analyzing other symptomatic chard plants in the field, since the discovery of AILV in chard occurred after the crop had been eradicated. 

This study highlighted the sequence variations in the RNA2 of AILV from different hosts and the presence of three stretches of amino acid sequences in the CP gene of the AILV-X87254 isolate that were significantly different from those found in our isolates. However, a comparison of the nucleotide sequence between this partial fragment (1820 nt long and including the CP and a portion of the 3′UTR region) and the sequences of the AILV isolates under study showed from 85% to 95% identity. It is hard to determine whether these variations were due to some sequencing errors of this partial fragment or to gene recombination events in this AILV isolate. In this context, our study predicted several different potential recombination sites involving the AILV genomes of different hosts, and, in particular, those of grapevine (AILV-V), artichoke (AILV-C), and chard (AILV-B). This recombination, and particularly in these host types, was almost expected, since these are plant species that often share the same agricultural habitats and in consideration of the fact that recombination events are quite frequent in nepoviruses [33]. It is likely that these recombinations are due to the existence of common vectors in nature, which could facilitate the encounter of different AILV isolates in the same plant. The existence of interspecific recombinants of AILV would provide indirect evidence for the existence of common nematode vectors capable of transmitting AILV to different host species, considering that, to date, the natural transmission of AILV by nematodes has not been experimentally proven for all known plant species. In fact, nematodes indiscriminately visiting different host species, from which they can acquire and/or transmit the virus, may have favored the “coexistence” of different isolates in the same plant and\or in infected seedlings, and consequently, possible gene recombinations among them. 

Experimental work to study the ability of *Longidorus attenuatus* and *L. apulus* to transmit AILV isolates from one host species to another could help to clarify the evolutionary pathway of this virus.

## Figures and Tables

**Table 1:** Nucleotide sequence Identity Matrix of 5′ (shadowed) and 3′ UTRs of AILV isolates.

Virus-Isolate	AILV-B	AILV-S	AILV-C	AILV-G	AILV-V
AILV-B	ID ^1^	98.9	94.6	96.2	85.2
AILV-S	98.2	ID	95.6	97.3	86.2
AILV-C	92.7	94.4	ID	97.3	87.5
AILV-G	92.7	94.4	100	ID	88.2
AILV-V	93.0	94.7	99.6	99.6	ID

^1^ ID: identical.

**Table 2:** Nucleotides (shadowed) and amino acids sequence Identity Matrix of RNA2 coding region of AILV isolates.

Virus-Isolate	AILV-B	AILV-S	AILV-C	AILV-G	AILV-V
AILV-B	ID ^1^	97.8	91.6	88.7	84.7
AILV-S	99	ID	93.7	90.7	86.4
AILV-C	88.5	89.3	ID	90	85.9
AILV-G	86.8	87.7	86.1	ID	90.1
AILV-V	82.3	83.1	82.2	86.2	ID

^1^ ID: identical.

**Table 3:** Nucleotides (shadowed) and amino acids sequence Identity Matrix of the CP of AILV isolates. AILV-X87254 is a partial sequence.

Virus-Isolate	AILV-B	AILV-S	AILV-C	AILV-G	AILV-V	AILV-X87254
AILV-B	ID ^1^	99	94.9	96.8	94.9	80.4
AILV-S	99.6	ID	95.8	97.8	95.7	81
AILV-C	88.1	88.4	ID	94.9	94.5	80
AILV-G	93.1	93.5	88.4	ID	94.7	80.2
AILV-V	88.6	88.9	87.3	89	ID	84.3
AILV-X87254	85.9	86.3	84.8	86.3	94.5	ID

^1^ ID: identical.

**Table 4:** Recombination crossover analysis of genomic RNA2 of AILV isolates, using the ‘Recombination Detecting Program’ RDP4 ^1^.

Domain	Isolate	Position (nt)	Parental Isolates (Major × Minor)	RDP4 (*p* Value) ^1^
2A	AILV-C	251-300	AILV-V × AILV-B	R**G**BMC3sS (4.516 × 10^−64^)
2A	AILV-G	730-800	AILV-S × AILV-V	**R**GBMC3sS (1.462 × 10^−67^)
2B	AILV-C	1292-1406	AILV-V × AILV-C	RG**B**MC3sS (1.124 × 10^−71^)
2B	AILV-C	1304-1408	AILV-V × AILV-B	RGBMC3s**S**(4.734 × 10^−38^)
2C	AILV-G	2245- 2851	AILV-B × AILV-V	RGBM**C**3sS (1.561 × 10^−27^)
2C	AILV-C	2762-2961	AILV-B × AILV-V	RGB**M**C3sS (1.053 × 10^−29^)
3′UTR	AILV-G	4450-4621	AILV-B × AILV-C	RGBMC**3s**S (2.573 × 10^−45^)

^1^ RDP4-implemented methods supporting the corresponding recombination sites: R (RDP), G (GENECONV), B (BOOTSCAN), M (MAXCHI), C (CHIMAERA), 3Seq (3s), and S (SISCAN). The P-value shown in brackets is the greatest *p*-value among those calculated using RDP4-implemented methods and the corresponding method is shown by underlined letters in bold type.

## Data Availability

Not applicable.

## Supplementary Materials

The following supporting information can be downloaded at: https://www.mdpi.com/article/10.3390/v15112170/s1, Table S1: List of primers used in 5’- and 3’-RACE and RT-PCR assays for amplifying and subsequent sequencing of genomic RNA2 of AILV isolates. V: degenerate nucleotide with A\C\G.

## References

[1] Cadman C.H. (1963). Biology of Soil-Borne Viruses. Ann. Rev. Phytopathol..

[2] Harrison B.D., Murant A.F. (1977). Nepovirus Group. CMI/AAB. Descriptions of Plant Viruses. Neth. J. Plant Pathol..

[3] Sanfacon H. (2015). *Secoviridae*: A Family of Plant Picorna-Like Viruses with Monopartite or Bipartite Genomes. eLS.

[4] (2023). ICTV. https://ictv.global.

[5] Majorana G., Rana G.L. (1970). Un nuovo virus latente isolato da Carciofo in Puglia/a new latent virus isolated from Artichoke in Apulia. Phytopathol Medit..

[6] Fuchs M., Schmitt-Keichinger C., Sanfaçon H. (2017). A renaissance in nepovirus research provides new insights into their molecular interface with hosts and vectors. Adv. Virus Res..

[7] Vovlas C., Martelli G.P., Quacquarelli A. (1971). Le virosi delle piante ortensi in Puglia. VI. II complesso delle maculature anulari della Cicoria. Phytopathol. Medit..

[8] Vovlas C. (1974). Le malformazioni fogliari, una nuova virosi del Geranio. Phytopathol. Medit..

[9] Quacquarelli A., Martelli G.P. Ricerche sull’agente dell’arricciamento maculato del carciofo. I. Ospiti differenziali e proprietà. Proceedings of the 1st Congr. Unione Fitopatol. Medit.

[10] Savino V., Gallitelli D., Jankulova M., Rana G.L. (1977). A comparison of four isolates of artichoke Italian latent virus (AILV). Phytopathol. Medit..

[11] Jankulova M., Savino V., Gallitelli D., Quacquarelli A., Martelli G.P. Isolation of artichoke Italian latent virus from the grapevine in Bulgaria. Proceedings of the 6th ICVG Meeting.

[12] Quacquarelli A., Rana G.L., Martelli G.P. (1976). Some weeds as host of pathogenic viruses in Apulia. Poljopr. Znan. Smotra.

[13] Elbeaino T., Belghacem I., Mascia T., Gallitelli D., Digiaro M. (2017). Next generation sequencing and molecular analysis of artichoke Italian latent virus. Arch. Virol..

[14] Le Gall O., Lanneau M., Candresse T., Dunez J. (1995). The nucleotide sequence of the RNA-2 of an isolate of the English serotype of tomato black ring virus: RNA recombination in the history of nepoviruses. J. General Virol..

[15] Vigne E., Marmonier A., Fuchs M. (2008). Multiple interspecies recombination events within RNA2 of Grapevine fanleaf virus and Arabis mosaic virus. Arch. Virol..

[16] Elbeaino T., Digiaro M., Gerbermeskel S., Martelli G.P. (2012). Grapevine deformation virus: Complete sequencing and evidence of recombination events derived from Grapevine fanleaf virus and Arabis mosaic virus. Virus Res..

[17] Digiaro M., Yahyaoui E., Martelli G.P., Elbeaino T. (2015). The sequencing of the complete genome of a Tomato black ring virus (TBRV) and of the RNA2 of three Grapevine chrome mosaic virus (GCMV) isolates from grapevine reveals the possible recombinant origin of GCMV. Virus Genes.

[18] Digiaro M., Nehdi S., Elbeaino T. (2012). Complete sequence of RNA1 of Grapevine Anatolian ringspot virus. Arch. Virol..

[19] Elbeaino T., Digiaro M., Fallanaj F., Kuzmanovic S., Martelli G.P. (2011). Complete nucleotides sequence and genome organization of Grapevine Bulgarian latent virus. Arch. Virol..

[20] Walker M., Chisholm J., Wei T., Ghoshal B., Saeed H., Rott M., Sanfaçon H. (2015). Complete genome sequence of three tomato ringspot virus isolates: Evidence for reassortment and recombination. Arch. Virol..

[21] Salleh W., Minutillo S.A., Spano R., Zammouri S., Gallitelli D., Mnari-Hattab M. (2017). Occurrence of artichoke-infecting viruses in Tunisia. EPPO Bull..

[22] Minutillo S.A., Mascia T., Gallitelli D. (2012). A DNA probe mix for the multiplex detection of ten artichoke viruses. Eur. J. Plant Pathol..

[23] Diener T.O., Schneider I.R. (1968). Virus degradation and nucleic acid release in single-phase phenol systems. Arch. Biochem. Biophys..

[24] Altschul S.F., Gish W., Miller W., Myers E.W., Lipman D.J. (1990). Basic local alignment search tool. J. Mol. Biol..

[25] Sanfaçon H. (2022). Re-examination of nepovirus polyprotein cleavage sites highlights the diverse specificities and evolutionary relationships of nepovirus 3C-like protease. Arch. Virol..

[26] Grieco F., Saponari M., Alkowni R., Savino V., Garau R., Martelli G.P. (2000). Progress in diagnosis of olive viruses. Infor. Fitopatol..

[27] Chen L., Brannigan K., Clark R., Gilbertson R.L. (2010). Characterization of curtoviruses associated with curly top disease of tomato in California and monitoring for these viruses in beet leafhoppers. Plant Dis..

[28] Nemchinov L.G., Hammond J., Jordan R., Hammond R.W. (2004). The complete nucleotide sequence, genome organization, and specific detection of Beet mosaic virus. Arch. Virol..

[29] Kundu K., Rysánek P. (2004). Detection of beet yellows virus by RT-PCR and immunocapture RT-PCR in Tetragonia expansa and Beta vulgaris. Acta Virol..

[30] Sabokkhiz M.A., Jafarpour B., Shahriari Ahmadi F., Tarighi S.I. (2012). dentification of Turnip mosaic virus isolated from Canola in northeast area of Iran. Afr. J. Biotechnol..

[31] Chaouachi M., Fortabat M.N., Geldreich A., Yot P., Kerlan C., Kebdani N., Audeon C., Romaniuk M., Bertheau Y. (2007). An accurate real-time PCR test for the detection and quantification of cauliflower mosaic virus (CaMV): Applicable in GMO screening. Eur. Food Res. Technol..

[32] Fuchs M., Jean-Michel Hily J.M., Petrzik K., Sanfaçon H., Thompson J.R., van der Vlugt R., Wetzel T., ICTV Report Consortium (2022). ICTV Virus Taxonomy Profile: Secoviridae. J. Gen. Virol..

[33] Hily J.M., Poulicard N., Kubina J., Reynard J.S., Spilmont A.S., Fuchs M., Lemaire O., and Vigne E. (2021). Metagenomic analysis of nepoviruses: Diversity, evolution and identification of a genome region in members of subgroup A that appears to be important for host range. Arch. Virol..

