# Supplementary material for: Identification, Sequencing, and Molecular Analysis of RNA2 of Artichoke Italian Latent Virus Isolates from Known Hosts and a New Host Plant Species
Source: Viruses. 2023 Oct 28;15(11):2170. doi: 10.3390/v15112170 (PMC10675341; doi:10.3390/v15112170)
Supplement: Supplementary file 1 [file viruses-15-02170-s001.zip › viruses-2614641-supplementary.pdf]

viruses

MDPI

Supplementary Table S1. List of primers used in 5′- and 3′-RACE and RT-PCR assays for amplifying and subsequent sequencing of genomic RNA2 of AILV isolates. V: degenerate nucleotide with A\C\G.

Primers	Sequence (5′-3′)	Position	Amplicons size (bp)
**F1s**	AAATTGGATATGGACCTCAAG	915- 935	330
**F1a**	ATTCGGCTACTTCTAGCAATT	1244- 1224	
**F2s**	GAAAAGGTTTTGACCTATCCT	3301- 3321	420
**F2a**	ACTCAATGGCATAATCCAAAA	3720- 3700	
**RACE-a**	AAATATAAAAGCCAAACAAA	259- 239	260
**1-s**	GAAAAGAAATTTTCAAAAACCT	1-22	1533
**2-a**	TTCATCCTCCAACTGGTCAA	1533- 1514	
**2-s**	AACCCCCGCGGTCTGTTCACC	1458- 1479	1673
**3-a**	TGGCCCCCTTCTCTGTGGAAA	3152- 3132	
**3-s**	CGGATGTTACCACAACTGTGG	3036- 3056	1594
**oligodT**	TTTTTTTTTTTTTTTTTTTTV	4630	
